# Magnetic resonance enterography, small bowel ultrasound and colonoscopy to diagnose and stage Crohn’s disease: patient acceptability and perceived burden

**DOI:** 10.1007/s00330-018-5661-2

**Published:** 2018-08-20

**Authors:** Anne Miles, Gauraang Bhatnagar, Steve Halligan, Arun Gupta, Damian Tolan, Ian Zealley, Stuart A. Taylor, on behalf of the METRIC investigators, Susan Mallett, Rachel Baldwin-Cleland, Stuart Bloom, John Hamlin, Ailsa Hart, Antony Higginson, Ilan Jacobs, Sara McCartney, Steve Morris, Charles Murray, Andrew Plumb, Richard Pollok, Shonit Punwani, Laura Quinn, Manuel Rodriguez-Justo, Andrew Salter, Simon Travis, Alastair Windsor, Peter Wylie, Jade Dyer, Pranitha Veeramalla, Steve Hibbert, Richard Ellis, Fergus Thursby-Pelham, Richard Beable, Nicola Gibbons, Claire Ward, Anthony O’Connor, Hannah Lambie, Rachel Hyland, Nigel Scott, Roger Lapham, Doris Quartey, Deborah Scimshaw, Helen Bungay, Maggie Betts, Simona Fourie, Niall Power, Rajapandian Ilangovan, Uday Patel, Evgenia Mainta, Phillip Lung, Ian Johnston, Mani Naghibi, Morgan Moorghen, Adriana Martinez, Francois Porte, Christopher Alexakis, James Pilcher, Anisur Rahman, Jonny Vlahos, Rebecca Greenhalgh, Anita Wale, Teresita Beeston, Wivijin Piga, Joey Clemente, Farooq Rahman, Simona de Caro, Shameer Metha, Rosa Vega, Roman Jastrub, Harbir Sidhu, Hameed Rafiee, Mairead Tennent, Caron Innes, Craig Mowat, Gillian Duncan

**Affiliations:** 1 Department of Psychological Sciences, Birkbeck, University of London, Malet Street, London, WC1E 7HX UK; 2 Centre for Medical Imaging, University College London, Charles Bell House, 43-45, Foley Street, London, W1W 7TS UK; 3 Intestinal Imaging Centre, St Mark’s Hospital, Harrow, UK; 4 Department of Radiology, St James’s University Hospital, Leeds Teaching Hospitals NHS Trust, Beckett Street, Leeds, LS9 7TF UK; 5 Department of Radiology, Ninewells Hospital, Dundee, Scotland DD1 9SY UK

**Keywords:** Magnetic resonance imaging, Ultrasound, Crohn disease, Patient preference, Patient satisfaction

## Abstract

**Objectives:**

To compare patient acceptability and burden of magnetic resonance enterography (MRE) and ultrasound (US) to each other, and to other enteric investigations, particularly colonoscopy.

**Methods:**

159 patients (mean age 38, 94 female) with newly diagnosed or relapsing Crohn’s disease, prospectively recruited to a multicentre diagnostic accuracy study comparing MRE and US completed an experience questionnaire on the burden and acceptability of small bowel investigations between December 2013 and September 2016. Acceptability, recovery time, scan burden and willingness to repeat the test were analysed using the Wilcoxon signed rank and McNemar tests; and group differences in scan burden with Mann–Whitney *U* and Kruskal–Wallis tests.

**Results:**

Overall, 128 (88%) patients rated MRE as very or fairly acceptable, lower than US (144, 99%; *p* < 0.001), but greater than colonoscopy (60, 60%; *p* < 0.001). MRE recovery time was longer than US (*p* < 0.001), but shorter than colonoscopy (*p* < 0.001). Patients were less willing to undergo MRE again than US (127 vs. 133, 91% vs. 99%; *p =* 0.012), but more willing than for colonoscopy (68, 75%; *p =* 0.017). MRE generated greater burden than US (*p* < 0.001), although burden scores were low. Younger age and emotional distress were associated with greater MRE and US burden. Higher MRE discomfort was associated with patient preference for US (*p =* 0.053). Patients rated test accuracy as more important than scan discomfort.

**Conclusions:**

MRE and US are well tolerated. Although MRE generates greater burden, longer recovery and is less preferred than US, it is more acceptable than colonoscopy. Patients, however, place greater emphasis on diagnostic accuracy than burden.

**Key Points:**

*• MRE and US are rated as acceptable by most patients and superior to colonoscopy.*

*• MRE generates significantly greater burden and longer recovery times than US, particularly in younger patients and those with high levels of emotional distress.*

*• Most patients prefer the experience of undergoing US than MRE; however, patients rate test accuracy as more importance than scan burden.*

**Electronic supplementary material:**

The online version of this article (10.1007/s00330-018-5661-2) contains supplementary material, which is available to authorized users.

## Introduction

Cross-sectional imaging plays a crucial role in the diagnosis and follow-up of Crohn’s disease (CD), and is fundamental to determine disease extent, activity and complications [1]. Although many techniques are available, emphasis is placed on MR enterography (MRE) and small bowel ultrasound (US) given the potential detrimental effects of repeated ionising radiation exposure associated with CT [1, 2]. Meta-analyses suggest that MRE and US are largely equivalent in terms of accuracy, although most comparative studies to date have been relatively small and single site [3–5], and implementation has been governed largely by availability, local expertise and clinician preference [6, 7]. However the results of the METRIC trial, a large prospective multicentre diagnostic accuracy study, have shown that although both MRE and US have high accuracy for the extent and activity of small bowel Crohn’s disease, MRE is superior to US when tested in a national health service setting [8, 9].

Patient experience and acceptability will influence test utility. While MRE and US avoid radiation, they have their own specific attributes which may impact on tolerance. For example, patients must ingest large volumes of oral contrast for MRE, and US requires abdominal compression. Patients’ perceptions of test “burden” (levels of physical and psychological discomfort) can impact on compliance, even if the test is diagnostically superior to alternatives, as exemplified by low uptake of colorectal cancer screening colonoscopy [10]. This diminishes test utility. Indeed, patients may delay seeking medical attention, fearing the discomfort associated with procedures such as colonoscopy [11]. To date little data reports imaging test preferences amongst patients with CD, and available data largely compares now largely obsolete investigations such as barium enema and enteroclysis [1].

The purpose of our study was to compare the perceived burden and acceptability of MRE to US, and to other enteric investigations in patients recruited to the METRIC trial [8], to identify predictors of scan preference and to determine the perceived importance of different scan attributes.

## Materials and methods

### Participants

**Fig. 1:**
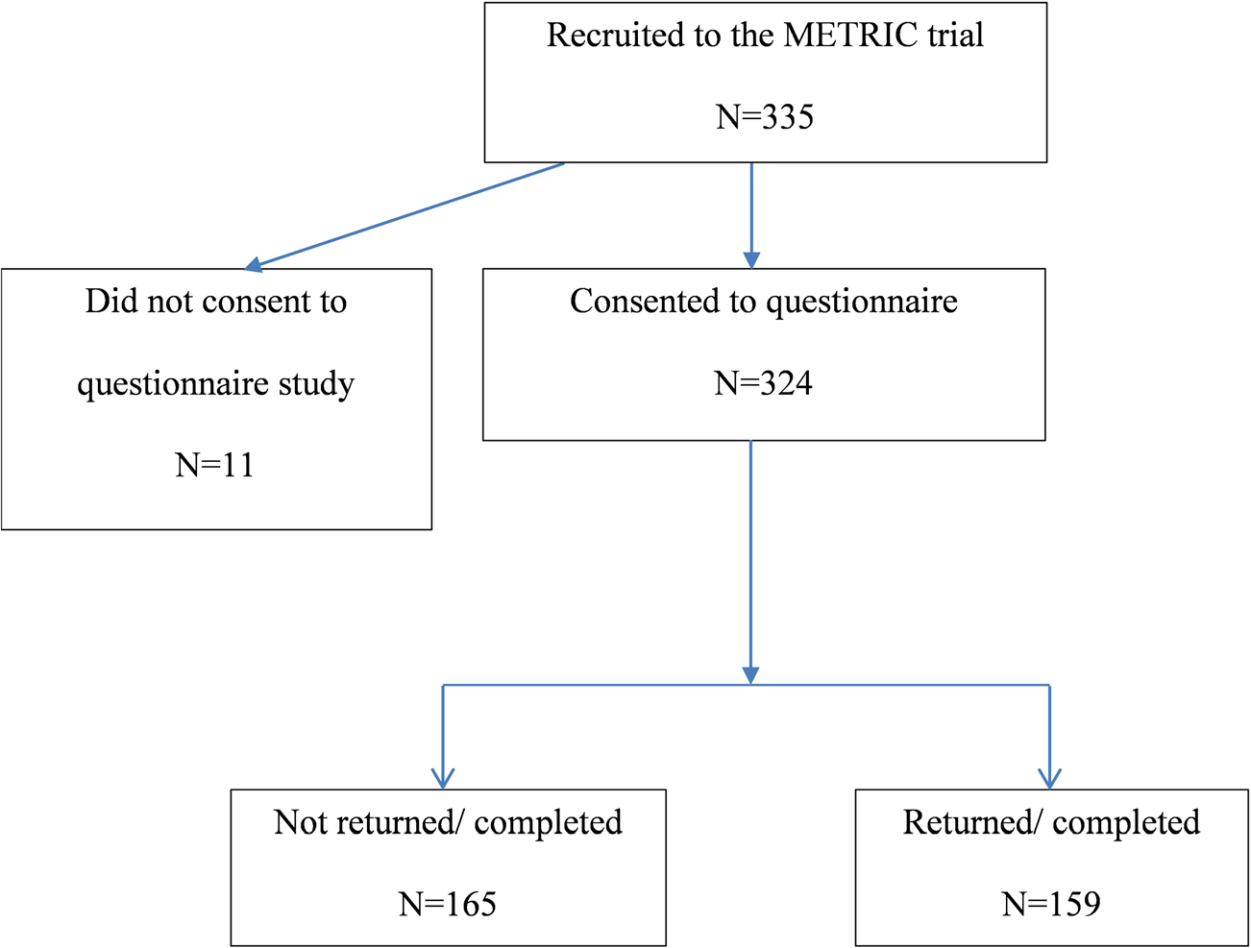
Flow chart showing flow of participants in study

The METRIC trial protocol was published previously [8], and the main trial was recently reported [9]. In summary, patients with newly diagnosed CD, or with known CD and suspected luminal relapse, were prospectively recruited from eight hospitals and underwent MRE and US, in addition to other small bowel investigations performed as part of usual clinical care. Patients with newly diagnosed CD had already undergone colonoscopy or had this pending; patients with suspected relapse only underwent colonoscopy if clinically indicated. Overall, 335 patients were recruited and all were given the option to complete a patient experience questionnaire investigating their experience. In total 324 (97%) consented to take part in the experience substudy, of whom 159 completed the questionnaire (48% of total recruitment) (see Fig. 1).

### Questionnaire distribution

Patients were provided with paper copies of the questionnaire at the time of consent by a member of their local trial team, or these were posted if this was not possible. A stamped addressed envelope for return was provided and patients were asked to complete the questionnaire only after all their investigations were completed for that particular diagnostic episode. Patients were encouraged to contact their clinical team if they were unsure whether they had completed their current round of investigations. Participants were asked to record the date of questionnaire completion.

### Questionnaire content

*Demographics:* Patients were asked their age, gender, educational level and ethnicity. Missing demographic data on age and gender were supplied via the central trial database.

*Physical and emotional well-being:* Emotional distress was assessed using the General Health Questionnaire GHQ-12 [12]. An example item is, “In the last three months have you….been feeling unhappy and depressed”. Using the GHQ-12 binary coding method (0,0,1,1), a mean sum score was created ranging from 0 to 12. A score of 4 or higher is considered indicative of significant distress levels [13].

Co-morbidity was assessed by asking patients about their current and recent physical health and mental well-being. Patients were asked to report (“yes” or “no”) whether they had any of the following diseases: heart or vascular disease, diabetes, epilepsy, stroke, arthritis, asthma, mental or emotional disorder. There was also an option to provide details of other illness. A response of “yes” to any illness was coded and summed to form a dichotomous “co-morbidity” variable (“present” or “absent”), but mental or emotional disorder was omitted since this was captured by the GHQ-12.

### Scan recovery, overall acceptability and willingness to have again

The questionnaire was divided into sections pertaining to MRE, US, hydro-US (ultrasound performed following oral contrast administration), barium follow-through (BaFT), CTE enterography (CTE) and colonoscopy. Patients were asked to indicate whether they had undergone the test and, if so, complete the relevant sections, or otherwise to leave that section blank.

For each investigation, patients graded their recovery time on a 9-point scale ranging from “immediate” to “a week”. Data were collapsed into six categories for analysis (see “Results”).

**Fig. 2:**
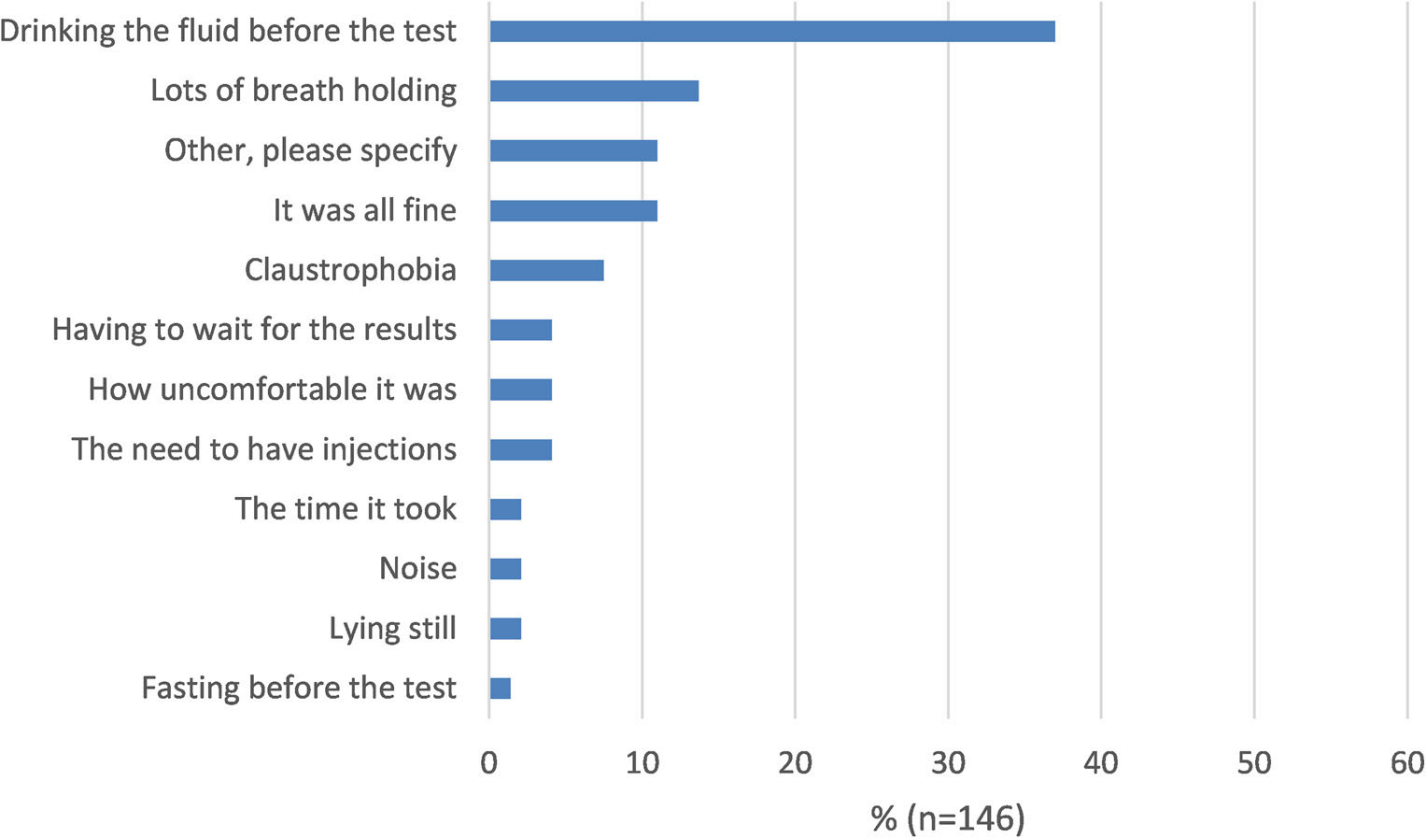
Comparative scan experience: least acceptable part of MR enterography

**Fig. 3:**
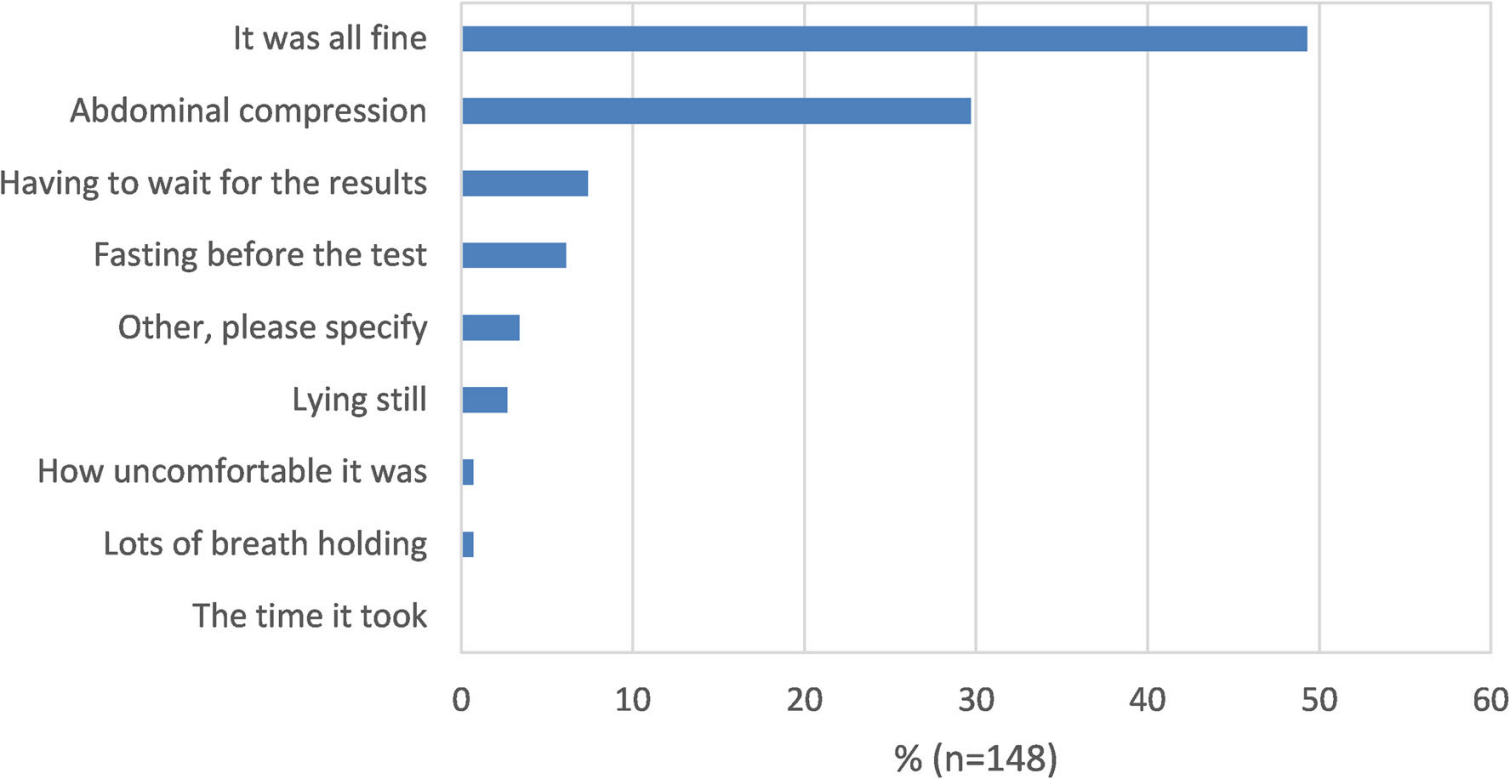
Comparative scan experience: least acceptable part of ultrasound

**Fig. 4:**
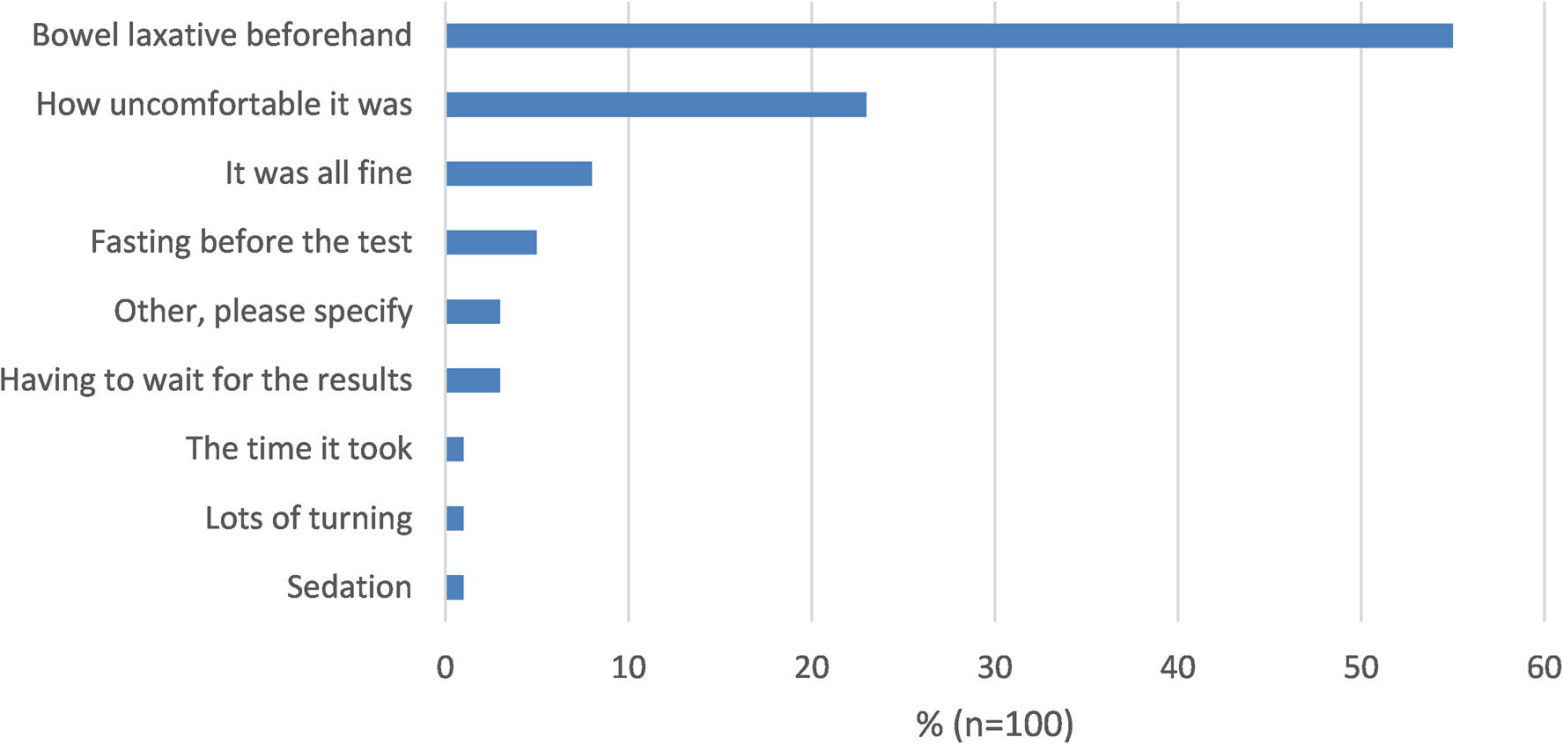
Comparative scan experience: least acceptable part of colonoscopy

Patients rated how acceptable they found investigations on a 4-point scale: “not at all acceptable” to “very acceptable” (see “Results”). Patients were also asked to select the least acceptable (or worst) part of the investigation from a range of attributes provided, specific to the particular investigation. For example, exposure to ionising radiation was listed as an option for CTE and BaFT, and laxative requirement listed for colonoscopy (see Figs. 2, 3 and 4, plus Supplementary Figs. 1–3). Patients were also asked if they would repeat the investigation if necessary (with response options “yes”, “no”, “not sure”).

### Scan burden for MRE and US

Scan burden for MRE and US was quantified using a questionnaire adapted from that used to assess colonoscopy and whole-body MRI [14, 15] (Supplementary Data 1 and 2). Five additional items of direct relevance to small bowel investigations were added: abdominal bloating, diarrhoea, nausea, vomiting and sleep difficulties. The questionnaire combined a series of individual items into three main domains: satisfaction, worry and discomfort. The MRE questionnaire included 31 items (7, 6 and 18 in satisfaction, worry and discomfort domains, respectively) and the US questionnaire included 28 items (7, 6 and 15 satisfaction, worry and discomfort domains, respectively), excluding items pertaining to noise, claustrophobia, injections and undesirable side effects, but additionally including an item relating to the abdominal pressure of the US probe.

Patients rated their experiences using a 1–7 Likert scale, where 1 and 7 were anchored to bipolar statements related to the scan, e.g. 1 = “the noise of the scanner was unbearable” to 7 = “the noise of the scanner was fine”. Scores for each item were reverse scored, totalled and averaged so that higher scores equated to higher burden. Internal reliability of subscales was assessed using Cronbach’s alpha.

### Scan preference

Patients were then asked to indicate whether they would prefer MRE or US if they had to undergo just one test.

### Overall perceived importance of investigation attributes

Patients were asked to rate how important 25 possible investigation attributes were to them on a 5-point scale: “not at all important” to “extremely important” (Supplementary Data 3). Higher scores indicated higher levels of perceived importance. Attributes included diagnostic accuracy and test efficiency to reach a final diagnosis as well as items specific to certain scans, for example requirement to drink a large volume of oral contrast.

### Statistical analysis

The study was powered to enable comparison of scan burden between MRE and US using the Wilcoxon signed rank test, with a medium effect size (*d* = 0.5), alpha of 0.05 and 95% power. A minimum number of 57 patients was required. Analysis was performed using IBM SPSS version 24 (IBM Corp.). Independent *t* tests and chi-square tests were used to assess differences between (i) questionnaire completers and non-completers and (ii) newly diagnosed and relapse cohorts, for continuous or categorical data respectively. Related samples Wilcoxon sign tests were used to assess differences between scan recovery time, scan acceptability and scan burden. McNemar tests were used to assess willingness to have the different investigations again. Bonferroni corrections were applied to the latter, meaning a *p* < 0.01 threshold for statistical significance. Differences in perceived MRE and US scan burden between different subgroups were assessed using Mann–Whitney *U* tests or Kruskal–Wallis as appropriate. Post hoc comparisons using a Bonferroni correction were used to assess the effect of age on scan burden, adopting a *p* < 0.01 threshold for statistical significance. The time between the questionnaire completion and the date of MRE and US was dichotomised into less than 5 weeks or 5 weeks or longer [16] and any association with scan preference or perceived importance of different test attributes assessed using chi-square tests and Spearman’s rho correlation coefficients respectively, adopting a *p* < 0.002 threshold following a Bonferroni correction (0.05/25). We also explored whether a time interval of less than or more than 1 week influenced scan preference.

## Ethical considerations

Ethical approval for the METRIC trial (including the current study) was obtained from the National Health Service Research Ethics Committee (NHS REC) in September 2013 (ref: 13/SC/0394).

## Results

Participant characteristics are shown in Table 1. Just under half of participants who consented to complete a questionnaire actually did so (159/ 324, 49%). Participants completing the questionnaire were significantly older than non-responders (mean age, 38.2 vs. 33.8 years; *t =* 2.603, *p =* 0.010), but there were no gender differences between groups (chi-square = 1.606, *p =* 0.205) or whether the patient was newly diagnosed or relapsing (chi-square = 1.763, *p =* 0.184).

**Table 1:** Participant characteristics

	All patients (*N* = 159)	New diagnosis (*n* = 84)	Relapse (*n* = 75)	Group differences
Age	38.2 (16.4)	37.3 (17.3)^a^	39.1 (15.4)^a^	*t* < 1, *p =* 0.484
Female gender	94 (59.1)	47 (56.0)^a^	47 (62.7)^a^	χ^2^ = 0.739, *p =* 0.390
Educational qualifications None Some Degree level or higher	8 (5.1) 91 (58.3) 57 (36.5)	4 (4.9)^b^ 45 (55.6) 32 (39.5)	4 (5.3)^a^ 46 (61.3) 25 (33.3)	χ^2^ = 0.641 *p =* 0.726
Ethnicity (white)	127 (92.0)	71 (93.4)^c^	56 (90.3)^c^	χ^2^ = 0.447, *p =* 0.504
Newly diagnosed	84 (52.8)^a^	–	–	–
Comorbidities (at least one comorbid illness)	65 (40.9)	35 (41.7)^a^	30 (40.0)^a^	χ^2^ = 0.046, *p =* 0.831
GHQ-12 (presence of high distress)	73 (48.3)	43 (51.2)^a^	30 (44.8)^c^	χ^2^ = 0.614, *p =* 0.433

Data are *n* (%) unless specified otherwise. Where there is missing data, percentage is valid %

^a^No missing data

^b^Missing data < 5%

^c^Missing data > 5%

There were no significant differences in demographics, educational level, ethnicity, presence of comorbidities or prevalence of significant psychological distress between those with newly diagnosed CD or suspected relapse. Overall, rates of psychological distress were high, with 48% reporting clinically significant levels (Table 1).

The median number of days between patients undergoing the MRE scan and completing the questionnaire was 7 (range 0–326; 46.6 weeks). The median number of days between patients undergoing the US scan and completing the questionnaire was 6 (range 0–326). The proportion who completed the questionnaire less than 5 weeks after their MRE and US scan was 61% (*n* = 70) and 61% (*n* = 71) respectively.

The number and percentage of patients who completed the questions about scan experience across the different imaging modalities are shown in Supplementary Table 1.

### Scan recovery, overall acceptability and willingness to have again

**Fig. 5:**
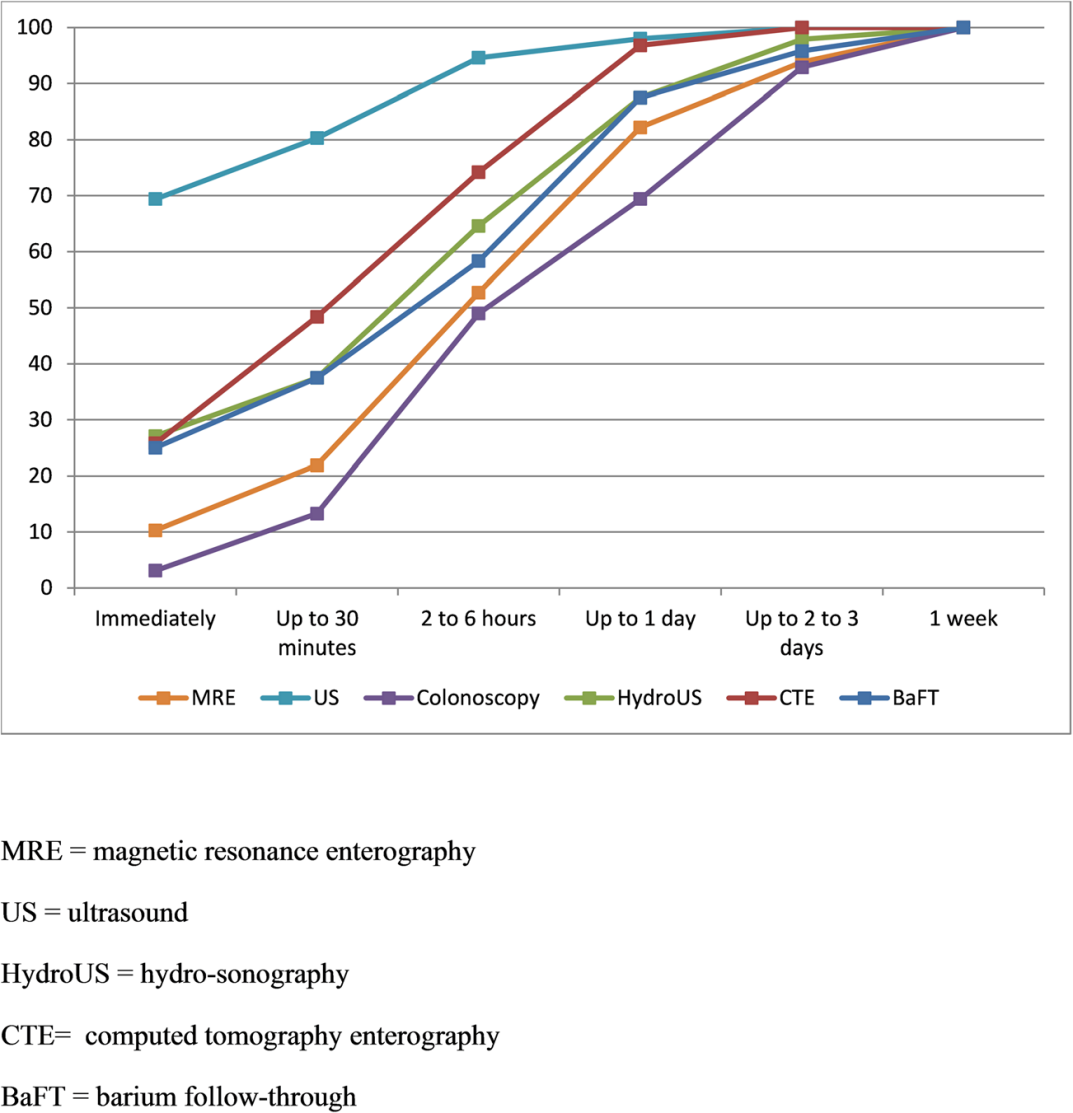
Recovery time by scan type (cumulative %)

MRE recovery time was significantly longer than US (*z =* 9.223, *p* < 0.001) but shorter than colonoscopy (*z =* 3.175, *p* < 0.001), with 15/146 (10%) reporting immediate recovery following MRE compared with 102/147 (69%) and 3/98 (3%) for US and colonoscopy respectively (Fig. 5, Supplementary Table 2). Overall 26/146 (18%) of patients took more than 1 day to recover from MRE, compared to 3/147 (2%) for US and 30/98 (31%) for colonoscopy. MRE recovery was also longer compared with CTE (*z =* 2.676, *p =* 0.007), but not different to BaFT (*z =* 0.645, *p =* 0.519). The difference between MRE and hydro-US approached significance (*z =* 2.245, *p =* 0.025) (Supplementary Table 2).

Overall, 128/145 (88%) of patients rated MRE as very or fairly acceptable. This was significantly lower than for US 144/146 (99%) (*z =* 6.696, *p* < 0.001), but significantly greater than for colonoscopy 60/100 (60%) (*z =* 4.480, *p* < 0.001). There were no differences in acceptability between MRE and hydro-US (*z =* 0.535, *p =* 0.593), BaFT (*z =* 1.567, *p =* 0.117) and CTE (*z =* 1.498, *p =* 0.134) (Table 2). Test acceptability for the new and relapsing cohorts were consistent with the findings for the whole cohort overall (Supplementary Tables 3 and 4).

**Table 2:** Comparative scan acceptability and willingness to have again (all participants)

	MR enterography	Ultrasound	Colonoscopy	Hydro-sonography	CTE	Barium follow-through
Acceptability	(*n* = 145)	(*n* = 146)	(*n* = 100)	(*n* = 46)	(*n* = 31)	(*n* = 24)
Very	66 (45.5)	126 (86.3)^b^	18 (18.0)^b^	28 (60.9)	17 (54.8)	8 (33.3)
Fairly	62 (42.8)	18 (12.3)	42 (42.0)	13 (28.3)	12 (38.7)	12 (50.0)
Slightly	12 (8.3)	0 (0)	34 (34.0)	4 (8.7)	2 (6.5)	1 (4.2)
Not at all	5 (3.4)	2 (1.4)	6 (6.0)	1 (2.2)	0 (0)	3 (12.5)
Willingness to have again	(*n* = 140)	(*n* = 135)	(*n* = 91)	(*n* = 42)	(*n* = 28)	(*n* = 22)
Yes	127 (90.7)	133 (98.5)^a^	68 (74.7)^a^	40 (95.2)	26 (92.9)	20 (90.9)
Not sure	12 (8.6)	0 (0)	14 (15.4)	2 (4.8)	1 (3.6)	1 (4.5)
No	1 (0.7)	2 (1.5)	9 (9.9)	0 (0)	1 (3.6)	1 (4.5)

Data are *n* (%). Where there is missing data, percentage is valid %

^a^Significantly different from MRE *p* < 0.05

^b^Significantly different from MRE *p* < 0.001

The proportion willing to repeat MRE was high (127/140, 91%), but lower than for US (133/135, 99%) (*p =* 0.012), which approached statistical significance at the *p* < 0.01 threshold. Patients were less willing to repeat colonoscopy (68/91, 75%) versus MRE (*p =* 0.017) and indeed 9/91 (10%) stated they would not be willing to undergo repeat colonoscopy. There were no significant differences between MRE and hydro-US (*p =* 0.219), CTE (*p =* 1.000), or BaFT (*p =* 1.000) (Table 2).

Attributes selected as the least acceptable part of MRE, US and colonoscopy are shown in Figs. 2 to 4 (see Supplementary Figs. 1–3 for hydro-US, BaFT and CTE). Drinking contrast (37%) and repeated breath-holding (14%) were most commonly cited for MRE, followed by “other” which comprised mainly side effects of drinking contrast, such as diarrhoea, pain and wind, feeling sick, abdominal soreness or bloating.

Overall 49% reported US as being “fine”, with no least acceptable part, although 30% reported abdominal compression as the least acceptable aspect. Conversely, for colonoscopy 55% of patients rated the laxative as the least acceptable part of the investigation, followed by discomfort (23%).

### Scan burden for MRE and US

Internal reliability of the subscales was good: for MRE the satisfaction subscale Cronbach’s alpha = 0.813, discomfort subscale Cronbach’s alpha = 0.872 and worry subscale Cronbach’s alpha = 0.786; for US the satisfaction subscale Cronbach’s alpha = 0.849, discomfort subscale Cronbach’s alpha = 0.868 and worry subscale Cronbach’s alpha = 0.808.

Burden scores for MRE and US are shown in Table 3. Patients reported higher burden during MRE versus US, although scores were relatively low overall. There were significant differences between MRE and US on all three subscales (discomfort: *z =* 9.558, *p* < 0.001; satisfaction: *z =* 7.043, *p* < 0.001; and worry: *z =* 8.017, *p* < 0.001).

**Table 3:** Perceived scan burden for MRE and US

Scan burden Low (1) to high (7)	MR enterography	Ultrasound	Wilcoxon signed rank test
Overall score	2.72 (0.96) (*n* = 148)	1.66 (0.74) (*n* = 149)	*z =* 9.536; *p* < 0.001
Discomfort subscale (1–7 = low to high)	3.01 (1.07) (*n* = 148)	1.65 (0.79) (*n* = 149)	*z =* 9.558; *p* < 0.001
Satisfaction subscale (1–7 = high to low)	2.07 (1.02) (*n* = 147)	1.49 (0.75) (*n* = 149)	*z =* 7.043; *p* < 0.001
Worry subscale (1–7 = low to high)	2.73 (1.23) (*n* = 148)	1.88 (1.08) (*n* = 149)	*z =* 8.017; *p* < 0.001

Data are means (SDs)

Differences in MRE scan burden according to patient demographics and scan preference are shown in Table 4. Perceived MRE scan burden was significantly higher among younger people (with significant differences only between the youngest and oldest age group, *z =* 2.969, *p =* 0.003 following Bonferroni corrections) and people with high levels of emotional distress. There was a non-significant trend towards higher MRE scan burden among patients reporting a preference for US. Younger age and high levels of emotional distress were also associated with higher perceived burden of US (see Table 4).

**Table 4:** Group differences in perceived MR enterography and ultrasound scan burden

	MR enterography burden	Group differences Mann–Whitney *U* or Kruskal–Wallis test	Ultrasound burden	Group differences Mann–Whitney *U* or Kruskal–Wallis test
Age		χ^2^ = 11.93, *p =* 0.008		χ^2^ = 8.93, *p =* 0.034
Up to 30	2.95 (0.89) (*n* = 61)		1.70 (0.60) (*n* = 61)	
30–49	2.72 (0.92)^a^ (*n* = 52)		1.66 (0.85) (*n* = 52)	
50–64	2.44 (1.12)^b^ (*n* = 22)		1.83 (0.95) (*n* = 21)	
65 and older	2.08 (0.83)^c^ (*n* = 13)		1.25 (0.30)^c^ (*n* = 15)	
Gender		*z =* 1.638, *p =* 0.101		*z =* 1.268, *p =* 0.205
Men	2.56 (0.90) (*n* = 61)		1.57 (0.70) (*n* = 62)	
Women	2.83 (0.98) (*n* = 87)		1.72 (0.77) (*n* = 87)	
Newly diagnosed or relapsing		*z =* 0.597, *p =* 0.550		*z =* 1.099, *p =* 0.272
Newly diagnosed	2.77 (0.97) (*n* = 81)		1.69 (0.71) (*n* = 82	
Relapsing	2.65 (0.95) (*n* = 67)		1.62 (0.78) (*n* = 67)	
Comorbidities		*z =* 0.279, *p =* 0.780		*z =* 0.481, *p =* 0.630
No	2.69 (0.93) (*n* = 85)		1.64 (0.68) (*n* = 84)	
Yes	2.76 (1.00) (*n* = 63)		1.68 (0.81) (*n* = 65)	
GHQ-12		*z =* 2.334, *p =* 0.020		*z =* 2.734, *p =* 0.006
Low distress	2.51 (0.92) (*n* = 74)		1.53 (0.73) (*n* = 76)	
High distress	2.91 (0.94) (*n* = 73)		1.80 (0.74) (*n* = 72)	
Scan preference		*z =* 1.934, *p =* 0.053		*z =* 0.156, *p =* 0.876
MRE	2.43 (0.99) (*n* = 25)		1.82 (1.03) (*n* = 25)	
US	2.81 (0.92) (*n* = 99)		1.63 (0.69) (*n* = 99)	

^a^Significantly different from age 65+ at *p* < 0.05

^b^Significantly different from age up to 30 at *p* < 0.05

^c^Significantly different from age up to 30 at *p* < 0.01

### Scan preference

When asked which scan patients would prefer, the majority who expressed a preference (100/125 [80%]) selected US over MRE. Scan preference was not related to the time between questionnaire completion and undergoing US (either less than 5 weeks vs. 5 weeks or longer, or less than 1 vs. 1 week or longer), χ^2^ = 2.733, *p =* 0.098 and χ^2^ = 0.901, *p =* 0.343 respectively. There was also no association between the time of questionnaire completion and undergoing MRE (less than 5 weeks vs. 5 weeks or longer χ^2^ = 2.421, *p =* 0.120, or less than 1 vs. 1 week or longer χ^2^ = 2.182, *p =* 0.140 respectively).

### Overall perceived importance of investigation attributes

**Fig. 6:**
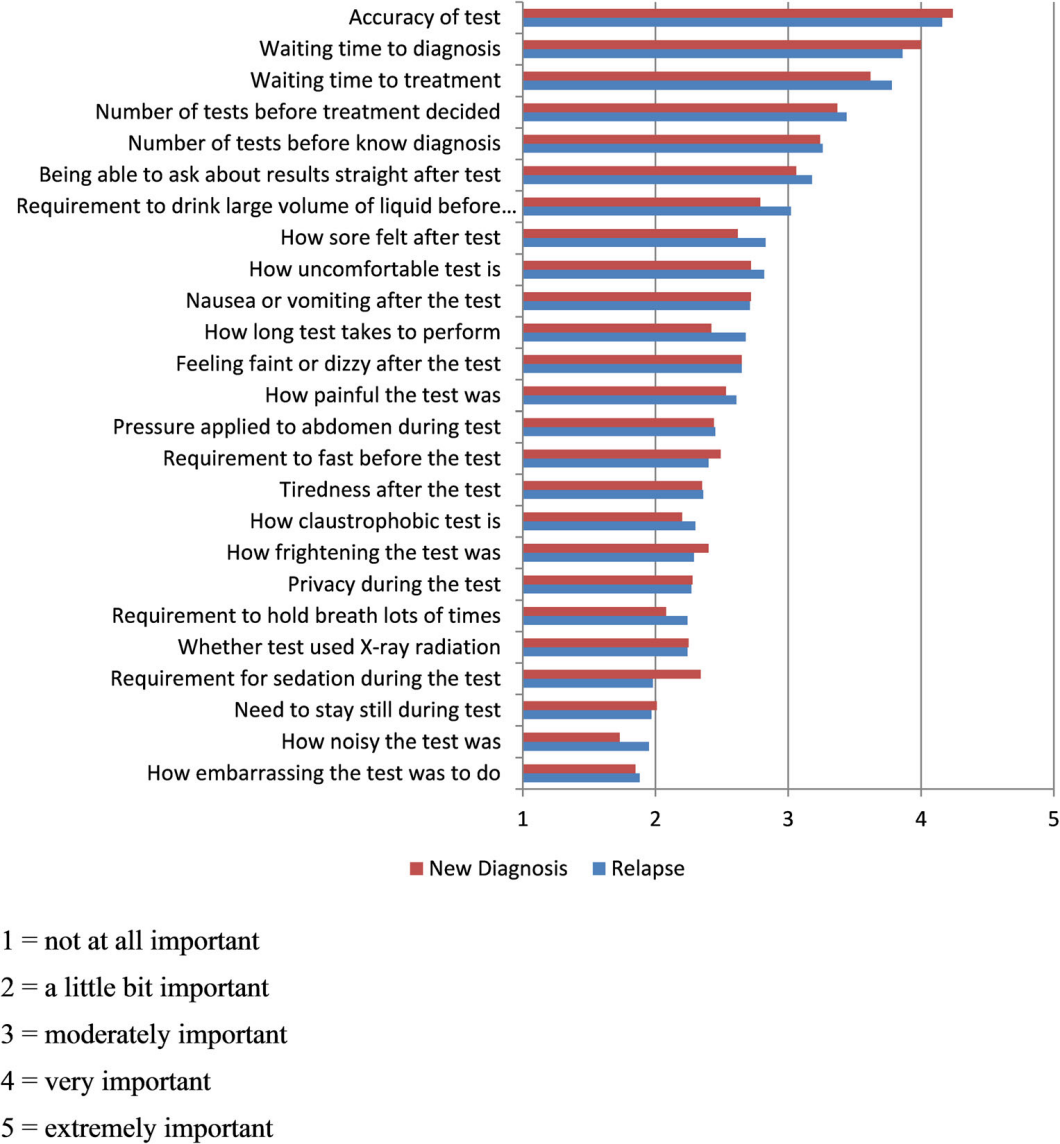
Perceived importance of different scan attributes (mean scores on a scale of 1–5)

Ratings of test attribute importance (graded from 1 = not at all important to 5 = extremely important) split according to patient cohort are shown in Fig. 6. For both cohorts, accuracy was rated as the most important attribute, followed by waiting time to diagnosis/ treatment and number of tests needed prior to final diagnosis. In general, negative physical test attributes such as requirement to drink fluid, test discomfort and fasting were rated as less important and generally between “a little bit important” and “moderately important”.

There was some evidence that, compared to those completing questionnaires within 5 weeks of MRE or US, patients completing questionnaires more than 5 weeks after perceived several physical attributes as less important (see Supplementary Table 5). However, none of these associations were significant following Bonferroni corrections, (*p* < 0.002).

## Discussion

Using questionnaire data from a large number of patients prospectively recruited to a diagnostic accuracy study, we found that MRE and US are both acceptable and reasonably well tolerated; most indicated that they would repeat the tests and scan burden scores were relatively low for both overall. However, MRE was judged significantly less favourably than US in terms of recovery time, acceptability, burden (across satisfaction, worry and discomfort domains) and “willingness to have again”, the last of these approaching significance at the *p* < 0.01 threshold. Putting this into context, MRE was still rated significantly more favourably than colonoscopy, which was the least acceptable of all tests. As could be anticipated, the “worse part” of the MRE scan was drinking enteric contrast beforehand and the associated side effects such as diarrhoea and/or abdominal pain/bloating, while conversely almost half indicated that US was “all fine”, with a minority listing abdominal compression as the worst part.

While our primary focus was MRE and US, we collected data on other small bowel investigations performed as part of usual clinical care in recruited patients. Recovery time for MRE was significantly longer than for CTE. However, we found no significant difference in recovery time compared to BaFT. This finding may be secondary to lack of statistical power contingent on small numbers undergoing BaFT, but it is possible that the constipating effects of barium contributed to slower recovery.

In general, MRI is a challenging test for patients. Relatively long scan times, claustrophobic scanner bore and associated noise all influence patient experience negatively [17]. Using a very similar questionnaire to the present study in a sample of 115 patients [15], patient burden during whole-body MRI (WB-MRI) cancer staging was reported as actually a little better than we found for MRE (2.21 vs. 2.72) [15]. Interestingly, both studies reported that high levels of emotional distress predicted increased MRI burden, although the current study found that younger age was also associated. Indeed, a very large proportion of participants in the current study reported significant psychological distress, comparable with high rates of anxiety recently among patients with active CD [18], and reaching levels more typically reported by patients being investigated for suspected cancer [19].

It is perhaps unsurprising that most patients stated that they would choose US over MRE, and indeed MRI scans have been judged to be more challenging than other scans such as PET-CT and contrast-enhanced spectral mammography [20, 21]. One very important finding from the current study is that patients rated several scan attributes as more important than the challenges and discomfort of undergoing scans. Notably, diagnostic accuracy was the most important attribute. This is comparable to data from studies of CT colonography [22]. Patients, at least to some extent, seem tolerant of discomfort if they believe that the test is more accurate than a less arduous alternative. We did not provide differential accuracy data for the tests under investigation, and it is likely that patients assumed they are similar when selecting preferences. The METRIC study has recently reported [9] and shows that when compared prospectively, MRE has significantly higher sensitivity for extent (presence and location) of small bowel Crohn’s disease than US (80% vs. 70%). When viewed in this context, and given their emphasis on diagnostic accuracy, it seems that MRE is an acceptable first-line test for patients; although patients’ experience during MRE was inferior to US, absolute levels of scan burden were relatively low and acceptability ratings reasonable. The performance of US in the METRIC trial was, however, still good, particularly for small bowel disease presence, and the technique still undoubtedly has a major role in managing Crohn’s disease patients. It is clearly a very well tolerated test by patients and completely safe, an important attribute given the potential deleterious effect of gadolinium deposition with repeated MRE [23–25]. Perhaps surprisingly, patients did not rate radiation exposure as particularly important, although again this may be influenced by their knowledge of this issue.

A very important consideration in questionnaire studies is the timing of the survey post intervention [26]. In a similar study comparing patient preferences for CT colonography verses colonoscopy, van Gelder et al reported that patient preference for CT colonography fell from 71% immediately after the tests to 61% 5 weeks later, and that drivers for preference switched from physical discomfort to relative diagnostic accuracy [16]. In the current study, we instructed patients to complete questionnaires after all their diagnostic tests had been completed rather than using a fixed time point [27]. The median return time in the current study was 1 week, although this ranged widely from 0 to 47 weeks. There was some evidence that after 5 weeks the perceived importance of a few attributes related to scan discomfort declined over time (although no significance was found after statistical correction). The rating of diagnostic accuracy as the priority for patients was not influenced by the time between the tests and questionnaire response, nor was overall patient scan preference, suggesting that our findings are robust.

Overall, it is clear therefore that the choice of imaging investigation should be based on a discussion between the referring clinician, radiologist and patient, considering scan attributes including diagnostic accuracy, patient experiences and priorities, and the exact underlying clinical question.

This study has limitations. Although the largest prospective study of patients’ experiences of cross-sectional imaging in Crohn’s disease to date, questionnaire response rates were under 50% despite the large majority stating that they would participate initially. However, this is consistent with questionnaire studies of similar design (e.g. [15, 28]). Non-responders were significantly younger than those who completed questionnaires, which may restrict generalisability. Postcode data were unavailable so we were unable to examine the influence of deprivation on questionnaire completion rates or scan burden/preference.

Since patients had already consented to the METRIC trial, the cohort sampled were apparently willing to undergo these tests in the first place. It would have been interesting to question those declining participation as to whether prior experience of either test had influenced their decision. We did not specifically record the experience of patients who did not complete or interrupted their imaging examination which would have been informative. In addition, some patients did not complete the questionnaire until weeks after their scan, and their recall of scan experiences may be imperfect. However, as noted above, the effect of such delay on reported experiences did not impact patient preferences. Finally, we used a variety of questionnaires, which, although comprehensive, may not fully capture the subtleties of patient experience.

In summary, both MRE and US are well tolerated generally by patients with CD, and better than colonoscopy. However, patient burden and recovery are significantly inferior for MRE compared to US. Whilst a majority of patients would opt to undergo US rather than MRE, patients rate other scan attributes, notably diagnostic accuracy, as more important than discomfort.

## Electronic supplementary material

ESM 1 (DOCX 58 kb)

## Abbreviations

BaFT: Barium follow-through
CD: Crohn’s disease
CTE: Computed tomography enterography
HydroUS: Hydro-sonography
MRE: Magnetic resonance enterography
MRI: Magnetic resonance imaging
US: Ultrasound
WB-MRI: Whole-body MRI

## References

[1] Panes J, Bouhnik Y, Reinisch W (2013). Imaging techniques for assessment of inflammatory bowel disease: joint ECCO and ESGAR evidence-based consensus guidelines. J Crohns Colitis.

[2] Desmond AN, O'Regan K, Curran C (2008). Crohn's disease: factors associated with exposure to high levels of diagnostic radiation. Gut.

[3] Dong J, Wang H, Zhao J (2014). Ultrasound as a diagnostic tool in detecting active Crohn's disease: a meta-analysis of prospective studies. Eur Radiol.

[4] Puylaert CA, Tielbeek JA, Bipat S, Stoker J (2015). Grading of Crohn's disease activity using CT, MRI, US and scintigraphy: a meta-analysis. Eur Radiol.

[5] Zhu C, Ma X, Xue L (2016). Small intestine contrast ultrasonography for the detection and assessment of Crohn disease: a meta-analysis. Medicine (Baltimore).

[6] Panes J, Bouzas R, Chaparro M (2011). Systematic review: the use of ultrasonography, computed tomography and magnetic resonance imaging for the diagnosis, assessment of activity and abdominal complications of Crohn's disease. Aliment Pharmacol Ther.

[7] Hafeez R, Greenhalgh R, Rajan J (2011). Use of small bowel imaging for the diagnosis and staging of Crohn's disease–a survey of current UK practice. Br J Radiol.

[8] Taylor S, Mallett S, Bhatnagar G (2014). METRIC (MREnterography or ulTRasound in Crohn's disease): a study protocol for a multicentre, non-randomised, single-arm, prospective comparison study of magnetic resonance enterography and small bowel ultrasound compared to a reference standard in those aged 16 and over. BMC Gastroenterol.

[9] Taylor SA, Mallett S, Bhatnagar G et al (2018) Diagnostic accuracy of magnetic resonance enterography and small bowel ultrasound for the extent and activity of newly diagnosed and relapsed Crohn’s disease (METRIC): a multicentre trial. Lancet Gastroenterol Hepatol S2468-1253(18)30161–4. 10.1016/S2468-1253(18)30161-4 10.1016/S2468-1253(18)30161-4 PMC6278907 29914843

[10] Plumb AA, Ghanouni A, Rainbow S et al (2017) Patient factors associated with non-attendance at colonoscopy after a positive screening faecal occult blood test. J Med Screen 24:12-19 10.1177/0969141316645629 27216771

[11] Casati J, Toner BB, de Rooy EC, Drossman DA, Maunder RG (2000). Concerns of patients with inflammatory bowel disease: a review of emerging themes. Dig Dis Sci.

[12] Goldberg D, Williams P (1988). A user's guide to the General Health Questionnaire.

[13] Knott C (2013) General mental and physical health. In: Craig R, Mindell J (eds). Health survey for England 2012, health social care and lifestyle. NatCen Social Research, London. http://content.digital.nhs.uk/catalogue/PUB13218/HSE2012-Ch4-Gen-health.pdf. Accessed 12 Feb 2018

[14] Salmon P, Shah R, Berg S, Williams C (1994). Evaluating customer satisfaction with colonoscopy. Endoscopy.

[15] Evans REC, Taylor SA, Beare S (2018). Perceived patient burden and acceptability of whole body MRI for staging lung and colorectal cancer; comparison with standard staging investigations. Br J Radiol.

[16] van Gelder RE, Birnie E, Florie J (2004). CT colonography and colonoscopy: assessment of patient preference in a 5-week follow-up study. Radiology.

[17] Evans R, Taylor S, Janes S (2017). Patient experience and perceived acceptability of whole-body magnetic resonance imaging for staging colorectal and lung cancer compared with current staging scans: a qualitative study. BMJ Open.

[18] Gracie DJ, Williams CJ, Sood R (2017). Negative effects on psychological health and quality of life of genuine irritable bowel syndrome-type symptoms in patients with inflammatory bowel disease. Clin Gastroenterol Hepatol.

[19] Brocken P, Prins JB, Dekhuijzen PN, van der Heijden HF (2012). The faster the better?—A systematic review on distress in the diagnostic phase of suspected cancer, and the influence of rapid diagnostic pathways. Psychooncology.

[20] Shortman RI, Neriman D, Hoath J (2015). A comparison of the psychological burden of PET/MRI and PET/CT scans and association to initial state anxiety and previous imaging experiences. Br J Radiol.

[21] Hobbs MM, Taylor DB, Buzynski S, Peake RE (2015). Contrast-enhanced spectral mammography (CESM) and contrast enhanced MRI (CEMRI): patient preferences and tolerance. J Med Imaging Radiat Oncol.

[22] von Wagner C, Halligan S, Atkin WS, Lilford RJ, Morton D, Wardle J (2009). Choosing between CT colonography and colonoscopy in the diagnostic context: a qualitative study of influences on patient preferences. Health Expect.

[23] Conte G, Preda L, Cocorocchio E (2017). Signal intensity change on unenhanced T1-weighted images in dentate nucleus and globus pallidus after multiple administrations of gadoxetate disodium: an intraindividual comparative study. Eur Radiol.

[24] Bae S, Lee HJ, Han K (2017). Gadolinium deposition in the brain: association with various GBCAs using a generalized additive model. Eur Radiol.

[25] Stojanov DA, Aracki-Trenkic A, Vojinovic S, Benedeto-Stojanov D, Ljubisavljevic S (2016). Increasing signal intensity within the dentate nucleus and globus pallidus on unenhanced T1W magnetic resonance images in patients with relapsing-remitting multiple sclerosis: correlation with cumulative dose of a macrocyclic gadolinium-based contrast agent. gadobutrol. Eur Radiol.

[26] Bjertnaes OA (2012). The association between survey timing and patient-reported experiences with hospitals: results of a national postal survey. BMC Med Res Methodol.

[27] Jensch S, Bipat S, Peringa J (2010). CT colonography with limited bowel preparation: prospective assessment of patient experience and preference in comparison to optical colonoscopy with cathartic bowel preparation. Eur Radiol.

[28] Miles A, Voorwinden S, Chapman S, Wardle J (2008). Psychologic predictors of cancer information avoidance among older adults: the role of cancer fear and fatalism. Cancer Epidemiol Biomarkers Prev.

