# Supplementary material for: Magnetic resonance enterography, small bowel ultrasound and colonoscopy to diagnose and stage Crohn’s disease: patient acceptability and perceived burden
Source: Eur Radiol. 2018 Aug 20;29(3):1083–93. doi: 10.1007/s00330-018-5661-2 (PMC6510862; doi:10.1007/s00330-018-5661-2)
Supplement: Supplementary file 1 — (DOCX 58 kb) [file 330_2018_5661_MOESM1_ESM.docx]

**Supplementary Figure 1: Comparative scan experience: least acceptable part of Hydro-sonography**

**Supplementary Figure 2: Comparative scan experience: least acceptable part of Barium follow-through**

**Supplementary Figure 3: Comparative scan experience: least acceptable part of CT**

**Supplementary data 1: MR Enterography scan burden questionnaire**

| **THINKING ABOUT THE SMALL BOWEL MRI THAT YOU HAD** (In this scan you were given a large drink beforehand. The scan takes about half an hour and you lie down in a tube (MRI scanner) sometimes with headphones on) | | | | | | | | |
| --- | --- | --- | --- | --- | --- | --- | --- | --- |
| **For each word or statement below, please tick** (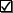) **the box on the scale which best describes your experience of the test. Please begin by looking at the example below.** | | | | | | | | |
| **EXAMPLE** | | | | | | | | |
| FOR EXAMPLE: If you felt that **time went slowly** you might tick the scale like this | | | | | | | | |
| Time went slowly | 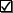 | □ | □ | □ | □ | □ | □ | Time went quickly |
| Or if you felt that **time went neither slowly nor quickly** you might tick the scale like this | | | | | | | | |
| Time went slowly | □ | □ | □ |  | □ | □ | □ | Time went quickly |
| **Now please answer the following** | | | | | | | | |
| Felt out of control | □ | □ | □ | □ | □ | □ | □ | Felt in control |
| Worried | □ | □ | □ | □ | □ | □ | □ | Not worried |
| Uncomfortable | □ | □ | □ | □ | □ | □ | □ | Comfortable |
| I was not interested | □ | □ | □ | □ | □ | □ | □ | I was interested |
| Loss of modesty | □ | □ | □ | □ | □ | □ | □ | No loss of modesty |
| The noise of the scanner unbearable | □ | □ | □ | □ | □ | □ | □ | The noise of the scanner was fine |
| Difficult to do what was required | □ | □ | □ | □ | □ | □ | □ | Easy to do what was required |
| Agitated | □ | □ | □ | □ | □ | □ | □ | Calm |
| Not confident in staff | □ | □ | □ | □ | □ | □ | □ | Confident in staff |
| Tired afterwards | □ | □ | □ | □ | □ | □ | □ | Not tired afterwards |
| The need to repeatedly hold my breath was unbearable | □ | □ | □ | □ | □ | □ | □ | The need to repeatedly hold my breath was fine |
| A bad experience | □ | □ | □ | □ | □ | □ | □ | A good experience |
| I was not pleased with how it went | □ | □ | □ | □ | □ | □ | □ | I was pleased with how it went |
| The time the scan took was unbearable | □ | □ | □ | □ | □ | □ | □ | The time the scan took was fine |
| Claustrophobic | □ | □ | □ | □ | □ | □ | □ | Not claustrophobic |
| Did not understand what was happening | □ | □ | □ | □ | □ | □ | □ | Understood what was happening |
| I was worried about what they would find | □ | □ | □ | □ | □ | □ | □ | I was not worried about what they would find |
| I was confused | □ | □ | □ | □ | □ | □ | □ | I was not confused |
| I felt puzzled | □ | □ | □ | □ | □ | □ | □ | I did not feel puzzled |
| Undignified | □ | □ | □ | □ | □ | □ | □ | Dignified |
| The injections needed for the scan were unbearable | □ | □ | □ | □ | □ | □ | □ | The injections need for the scan were fine |
| Dissatisfied | □ | □ | □ | □ | □ | □ | □ | Satisfied |
| Undesirable side effects | □ | □ | □ | □ | □ | □ | □ | No undesirable side effects |
| Not enough privacy | □ | □ | □ | □ | □ | □ | □ | Enough privacy |
| Hard to cope with | □ | □ | □ | □ | □ | □ | □ | Easy to cope with |
| The need to lie still for the scan was unbearable | □ | □ | □ | □ | □ | □ | □ | The need to lie still for the scan was fine |
| Severe abdominal bloating | □ | □ | □ | □ | □ | □ | □ | No abdominal bloating |
| Severe diarrhoea | □ | □ | □ | □ | □ | □ | □ | No diarrhoea |
| Severe nausea | □ | □ | □ | □ | □ | □ | □ | No nausea |
| Severe vomiting | □ | □ | □ | □ | □ | □ | □ | No vomiting |
| Severe sleep difficulties | □ | □ | □ | □ | □ | □ | □ | No sleep difficulties |

**Supplementary data 2: Ultrasound scan burden questionnaire**

| **THINKING ABOUT THE SMALL BOWEL ULTRASOUND SCAN THAT YOU HAD** (In this scan you were NOT given a large drink beforehand. Afterwards the doctor puts jelly on your abdomen and images are taken using a probe on the skin) | | | | | | | | |
| --- | --- | --- | --- | --- | --- | --- | --- | --- |
| **For each word or statement below, please tick** (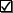) **the box on the scale which best describes your experience of the test. Please begin by looking at the example below.** | | | | | | | | |
| **EXAMPLE** | | | | | | | | |
| FOR EXAMPLE: If you felt that **time went slowly** you might tick the scale like this | | | | | | | | |
| Time went slowly | 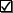 | □ | □ | □ | □ | □ | □ | Time went quickly |
| Or if you felt that **time went neither slowly nor quickly** you might tick the scale like this | | | | | | | | |
| Time went slowly | □ | □ | □ |  | □ | □ | □ | Time went quickly |
| **Now please answer the following** | | | | | | | | |
| Felt out of control | □ | □ | □ | □ | □ | □ | □ | Felt in control |
| Worried | □ | □ | □ | □ | □ | □ | □ | Not worried |
| Uncomfortable | □ | □ | □ | □ | □ | □ | □ | Comfortable |
| I was not interested | □ | □ | □ | □ | □ | □ | □ | I was interested |
| Loss of modesty | □ | □ | □ | □ | □ | □ | □ | No loss of modesty |
| The pressure of the probe was unbearable | □ | □ | □ | □ | □ | □ | □ | The pressure of the probe was fine |
| Difficult to do what was required | □ | □ | □ | □ | □ | □ | □ | Easy to do what was required |
| Agitated | □ | □ | □ | □ | □ | □ | □ | Calm |
| Not confident in staff | □ | □ | □ | □ | □ | □ | □ | Confident in staff |
| Tired afterwards | □ | □ | □ | □ | □ | □ | □ | Not tired afterwards |
| The need to repeatedly hold my breath was unbearable | □ | □ | □ | □ | □ | □ | □ | The need to repeatedly hold my breath was fine |
| A bad experience | □ | □ | □ | □ | □ | □ | □ | A good experience |
| I was not pleased with how it went | □ | □ | □ | □ | □ | □ | □ | I was pleased with how it went |
| The time the scan took was unbearable | □ | □ | □ | □ | □ | □ | □ | The time the scan took was fine |
| Did not understand what was happening | □ | □ | □ | □ | □ | □ | □ | Understood what was happening |
| I was worried about what they would find | □ | □ | □ | □ | □ | □ | □ | I was not worried about what they would find |
| I was confused | □ | □ | □ | □ | □ | □ | □ | I was not confused |
| I felt puzzled | □ | □ | □ | □ | □ | □ | □ | I did not feel puzzled |
| Undignified | □ | □ | □ | □ | □ | □ | □ | Dignified |
| Dissatisfied | □ | □ | □ | □ | □ | □ | □ | Satisfied |
| Not enough privacy | □ | □ | □ | □ | □ | □ | □ | Enough privacy |
| Hard to cope with | □ | □ | □ | □ | □ | □ | □ | Easy to cope with |
| The need to lie still for the scan was unbearable | □ | □ | □ | □ | □ | □ | □ | The need to lie still for the scan was fine |
| Severe abdominal bloating | □ | □ | □ | □ | □ | □ | □ | No abdominal bloating |
| Severe diarrhoea | □ | □ | □ | □ | □ | □ | □ | No diarrhoea |
| Severe nausea | □ | □ | □ | □ | □ | □ | □ | No nausea |
| Severe vomiting | □ | □ | □ | □ | □ | □ | □ | No vomiting |
| Severe sleep difficulties | □ | □ | □ | □ | □ | □ | □ | No sleep difficulties |

**Supplementary data 3: Questionnaire assessing perceived importance of different test attributes**

| Thinking about the investigations/ tests you have had - how IMPORTANT were the following things to you? | | | | | |
| --- | --- | --- | --- | --- | --- |
|  | Not at all important | A little bit important | Moderately important | Very important | Extremely important |
| The **number** of tests you needed to have before you knew what your full **diagnosis** was | □ | □ | □ | □ | □ |
| If there was more than one treatment, the **number** of tests you needed before doctors could choose the **best treatment** to help your condition/ cure your symptoms | □ | □ | □ | □ | □ |
| **How long** you had to wait to get a full **diagnosis** | □ | □ | □ | □ | □ |
| If there was more than one treatment, **how long** you had to wait because of tests before doctors could choose the **best treatment** to help your condition/ cure your symptoms | □ | □ | □ | □ | □ |
| How accurate the result of the test was (i.e. how good the test was in getting the full and correct diagnosis) | □ | □ | □ | □ | □ |
| How long the test took (for example 10 mins or 1 hour) | □ | □ | □ | □ | □ |
| How claustrophobic the test was | □ | □ | □ | □ | □ |
| How uncomfortable the test was | □ | □ | □ | □ | □ |
| If you had to hold your breath lots of times for the test | □ | □ | □ | □ | □ |
| If you had to lie very still for the test | □ | □ | □ | □ | □ |
| The need to prepare for the test by fasting (not eating) | □ | □ | □ | □ | □ |
| How embarrassing it was to do the test | □ | □ | □ | □ | □ |
| How painful it was to do the test | □ | □ | □ | □ | □ |
| How noisy the test was | □ | □ | □ | □ | □ |
| Whether you needed to be sedated (with drugs to make you sleepy) during the test | □ | □ | □ | □ | □ |
| How sore it made you after the test | □ | □ | □ | □ | □ |
| How much privacy you had during the test | □ | □ | □ | □ | □ |
| How tired you felt after the test | □ | □ | □ | □ | □ |
| Whether you suffered nausea/ vomiting after the test | □ | □ | □ | □ | □ |
| Whether you felt faint or dizzy after the test | □ | □ | □ | □ | □ |
| How frightening it was to do the test | □ | □ | □ | □ | □ |
| Whether you were exposed to X-Ray radiation as a result of having the test | □ | □ | □ | □ | □ |
| If you had to drink a large volume of liquid before the test | □ | □ | □ | □ | □ |
| If pressure was applied to your abdomen by the person during the test | □ | □ | □ | □ | □ |
| Being able to ask about the results straight after the test | □ | □ | □ | □ | □ |

**Supplementary Table 1**: **Participant response rates for the different scan experience items across modalities. Numbers are n (percent)**

|  | MR Enterography | Ultrasound | Colonoscopy | Hydro-sonography | CT | Barium follow-through |
| --- | --- | --- | --- | --- | --- | --- |
| Scan recovery time | 146 (91.8) | 147 (92.5) | 98 (61.6) | 48 (30.2) | 31 (19.5) | 24 (15.1) |
| Acceptability | 145 (91.2) | 146 (91.8) | 100 (62.9) | 46 (28.9) | 31 (19.5) | 24 (15.1) |
| Least acceptable part | 146 (91.8) | 148 (93.1) | 100 (62.9) | 48 (30.2) | 31 (19.5) | 23 (14.5) |
| Willingness to have again | 140 (88.1) | 135 (84.9) | 91 (57.2) | 42 (26.4) | 28 (17.6) | 22 (13.8) |

**Supplementary Table 2: Comparative scan recovery time (all participants) (numbers are n (%))**

|  | MR enterography | Ultrasound | Colonoscopy | Hydro-sonography | CT | Barium follow-through |
| --- | --- | --- | --- | --- | --- | --- |
| *Recovery time* | *(n=146)* | *(n=147)* | *(n=98)* | *(n=48)* | *(n=31)* | *(n=24)* |
| Immediate | 15 (10.3) | 102 (69.4)^c^ | 3 (3.1)^c^ | 13 (27.1)^a^ | 8 (25.8)^b^ | 6 (25.0) |
| Up to 30 minutes | 17 (11.6) | 16 (10.9) | 10 (10.2) | 5 (10.4) | 7 (22.6) | 3 (12.5) |
| Up to 2-6 hours | 45 (30.8) | 21 (14.3) | 35 (35.7) | 13 (27.1) | 8 (25.8) | 5 (20.8) |
| Up to 1 day | 43 (29.5) | 5 (3.4) | 20 (20.4) | 11 (22.9) | 7 (22.6) | 7 (29.2) |
| Up to 2-3 days | 17 (11.6) | 3 (2.0 ) | 23 (23.5) | 5 (10.4) | 1 (3.2) | 2 (8.3) |
| A week | 9 (6.2) | 0 (0) | 7 (7.1) | 1 (2.1) | 0 (0) | 1 (4.2) |

^a^ Significantly different from MRE p<0.05

^b^ Significantly different from MRE p<0.01

^c^ Significantly different from MRE p<0.001

**Supplementary Table 3: Comparative scan experience: newly diagnosed patients. Numbers are N (%).**

|  | MR enterography | Ultrasound | Colonoscopy |
| --- | --- | --- | --- |
| *Recovery time* | *(n=80)* | *(n=82)* | *(n=70)* |
| Immediate | 9 (11.3) | 54 (65.9) | 2 (2.9) |
| Up to 30 minutes | 10 (12.5) | 11 (13.4) | 7 (10.0) |
| Up to 2-6 hours | 21 (26.3) | 13 (15.9) | 28 (40.0) |
| Up to 1 day | 26 (32.5) | 3 (3.7) | 13 (18.6) |
| Up to 2-3 days | 9 (11.3) | 1 (1.2) | 14 (20.0) |
| A week | 5 (6.3) | 0 (0) | 6 (8.6) |
| *Acceptability* | *(n=80)* | *(n=81)* | *(n=70)* |
| Very | 41 (51.2) | 69 (85.2) | 15 (21.4) |
| Fairly | 31 (38.8) | 12 (14.8) | 29 (41.4) |
| Slightly | 6 (7.5) | 0 (0) | 22 (31.4) |
| Not at all | 2 (2.5) | 0 (0) | 4 (5.7) |
| *Willingness to have again* | *(n=76)* | *(n=71)* | *(n=64)* |
| Yes | 67 (88.2) | 70 (98.6) | 48 (75.0) |
| Not sure | 8 (10.5) | 0 (0) | 10 (15.6) |
| No | 1 (1.3) | 1 (1.4) | 6 (9.4) |

**Supplementary Table 4: Comparative scan experience: relapsing patients. Numbers are N (%)**

|  | MR enterography | Ultrasound | Colonoscopy |
| --- | --- | --- | --- |
| *Recovery time* | *(n=66)* | *(n=66)* | *(n=28)* |
| Immediate | 6 (9.1) | 48 (73.8) | 1 (3.6) |
| Up to 30 minutes | 7 (10.6) | 5 (7.7) | 3 (10.7) |
| Up to 2-6 hours | 24 (36.4) | 8 (12.3) | 7 (25.0) |
| Up to 1 day | 17 (25.8) | 2 (3.1) | 7 (25.0) |
| Up to 2-3 days | 8 (12.1) | 2 (3.1) | 9 (32.1) |
| A week | 4 (6.1) | 0 (0) | 1 (3.6) |
| *Acceptability* | *(n=65)* | *(n=65)* | (n=30) |
| Very | 25 (38.5) | 57 (87.7) | 3 (10.0) |
| Fairly | 31 (47.7) | 6 (9.2) | 13 (43.3) |
| Slightly | 6 (9.2) | 0 (0) | 12 (40.0) |
| Not at all | 3 (4.6) | 2 (3.1) | 2 (6.7) |
| *Willingness to have again* | *(n=64)* | (n=64) | *(n=27)* |
| Yes | 60 (93.8) | 63 (98.4) | 20 (74.1) |
| Not sure | 4 (6.3) | 0 (0) | 4 (14.8) |
| No | 0 (0) | 1 (1.6) | 3 (11.1) |

Supplementary Table 5: Correlations between time between scan and questionnaire completion and perceived importance of different scan attributes.

|  | Time between MRE and questionnaire completion (<5 weeks vs. 5 or more weeks) | Time between US and questionnaire completion completion (<5 weeks vs. 5 or more weeks) |
| --- | --- | --- |
| Accuracy of test | -0.071,  p=0.477,  N=102 | -0.114  P=0.249  N=104 |
| Requirement to hold breath lots of times | 0.072  P=0.465  N=105 | 0.048  P=0.626  N=107 |
| How claustrophobic test is | 0.033  P=0.737  N=105 | 0.013  P=0.893  N=107 |
| Requirement to drink large volume of liquid before test | -0.096  P=0.334  N=103 | -0.096  P=0.329  N=105 |
| How embarrassing the test was to do | -0.021  P=0.831  N=104 | 0.017  P=0.866  N=106 |
| Feeling faint or dizzy after the test | -0.218  P=0.026  N=105 | -0.216  P=0.025  N=107 |
| Requirement to fast before the test | 0.082  P=0.408  N=105 | 0.080  P=0.411  N=107 |
| How frightening the test was | -0.259  P=0.008  N=104 | -0.221  P=0.023  N=106 |
| How long test takes to perform | 0.040  P=0.691  N=103 | 0.048  P=0.629  N=105 |
| Number of tests before treatment decided | -0.126  P=0.206  N=103 | -0.107  P=0.277  N=105 |
| Number of tests before know diagnosis | -0.003  P=0.979  N=104 | -0.025  P=0.801  N=106 |
| Nausea or vomiting after the test | -0.204  P=0.037  N=105 | -0.192  P=0.047  N=107 |
| How noisy the test was | 0.038  P=0.700  N=104 | 0.024  P=0.810  N=106 |
| How painful the test was | -0.169  P=0.084  N=105 | -0.149  P=0.125  N=107 |
| Pressure applied to abdomen during test | -0.145  P=0.145  N=102 | -0.151  P=0.127  N=104 |
| Privacy during the test | -0.102  P=0.303  N=104 | -0.064  P=0.517  N=106 |
| Being able to ask about results straight after test | -0.013  P=0.895  N=103 | -0.026  P=0.795  N=105 |
| Requirement for sedation during the test | -0.017  P=0.864  N=101 | -0.007  P=0.944  N=103 |
| How sore felt after test | -0.259  P=0.008  N=104 | -0.279  P=0.004  N=106 |
| Tiredness after the test | -0.256  P=0.009  N=104 | -0.276  P=0.004  N=106 |
| How uncomfortable test is | -0.027  P=0.789  N=103 | -0.030  P=0.764  N=105 |
| Need to stay still during test | 0.063  P=0.521  N=105 | 0.039  P=0.690  N=107 |
| Waiting time to diagnosis | -0.001  P=0.992  N=103 | -0.036  P=0.719  N=105 |
| Waiting time to treatment | -0.055  P=0.610  N=87 | -0.058  P=0.592  N=89 |
| Whether test used X-ray radiation | -0.151  P=0.127  N=103 | -0.139  P=0.156  N=105 |
